# Re-attendance in supplemental breast MRI screening rounds of the DENSE trial for women with extremely dense breasts

**DOI:** 10.1007/s00330-024-10685-9

**Published:** 2024-04-19

**Authors:** Stefanie G. A. Veenhuizen, Sophie E. L. van Grinsven, Isabelle L. Laseur, Marije F. Bakker, Evelyn M. Monninkhof, Stéphanie V. de Lange, Ruud M. Pijnappel, Ritse M. Mann, Marc B. I. Lobbes, Katya M. Duvivier, Mathijn D. F. de Jong, Claudette E. Loo, Nico Karssemeijer, Paul J. van Diest, Wouter B. Veldhuis, Carla H. van Gils, DENSE Trial Study Group, C H van Gils, M F Bakker, S E L van Grinsven, S V de Lange, S G A Veenhuizen, W B Veldhuis, R M Pijnappel, M J Emaus, E M Monninkhof, M A Fernandez-Gallardo, M A A J van den Bosch, P J van Diest, R M Mann, R Mus, M Imhof-Tas, N Karssemeijer, C E Loo, P K de Koekkoek-Doll, H A O Winter-Warnars, R H C Bisschops, M C J M Kock, R K Storm, P H M van der Valk, M B I Lobbes, S Gommers, M B I Lobbes, M D F de Jong, M J C M Rutten, K M Duvivier, P de Graaf, J Veltman, R L J H Bourez, H J de Koning

**Affiliations:** 1 Julius Center for Health Sciences and Primary Care, University Medical Center Utrecht, Utrecht University, Stratenum 6.131, P.O. Box 85500, 3508 GA Utrecht, The Netherlands; 2 Department of Radiology, University Medical Center Utrecht, Utrecht University, P.O. Box 85500, 3508 GA Utrecht, The Netherlands; 3 Dutch Expert Centre for Screening, P.O. Box 6873, 6503 GJ Nijmegen, The Netherlands; 4 Department of Radiology, Radboud University Nijmegen Medical Center, P.O. Box 9101, 6500 HB Nijmegen, The Netherlands; 5 Department of Radiology, the Netherlands Cancer Institute - Antoni Van Leeuwenhoek Hospital, P.O. Box 90203, 1006 BE Amsterdam, The Netherlands; 6 Department of Radiology and Nuclear Medicine, Maastricht University Medical Centre, P.O. Box 5800, 6202 AZ Maastricht, The Netherlands; 7 Department of Medical Imaging, Zuyderland Medical Centre, P.O. Box 5500, 6130 MB Sittard-Geleen, The Netherlands; 8 GROW School for Oncology and Developmental Biology, Maastricht University, Maastricht, The Netherlands; 9 Department of Radiology, Jeroen Bosch Hospital, P.O. Box 90153, 5200 ME ‘S-Hertogenbosch, The Netherlands; 10 Department of Radiology and Nuclear Medicine, Cancer Center Amsterdam, Amsterdam UMC, Vrije Universiteit Amsterdam, P.O. Box 7057, 1007 MB Amsterdam, The Netherlands; 11 Department of Pathology, University Medical Center Utrecht, Utrecht University, P.O. Box 85500, 3508 GA Utrecht, The Netherlands

**Keywords:** Breast density, Breast neoplasms, Early detection of cancer, Magnetic resonance imaging, Patient participation

## Abstract

**Objectives:**

Supplemental MRI screening improves early breast cancer detection and reduces interval cancers in women with extremely dense breasts in a cost-effective way. Recently, the European Society of Breast Imaging recommended offering MRI screening to women with extremely dense breasts, but the debate on whether to implement it in breast cancer screening programs is ongoing. Insight into the participant experience and willingness to re-attend is important for this discussion.

**Methods:**

We calculated the re-attendance rates of the second and third MRI screening rounds of the DENSE trial. Moreover, we calculated age-adjusted odds ratios (ORs) to study the association between characteristics and re-attendance. Women who discontinued MRI screening were asked to provide one or more reasons for this.

**Results:**

The re-attendance rates were 81.3% (3458/4252) and 85.2% (2693/3160) in the second and third MRI screening round, respectively. A high age (> 65 years), a very low BMI, lower education, not being employed, smoking, and no alcohol consumption were correlated with lower re-attendance rates. Moderate or high levels of pain, discomfort, or anxiety experienced during the previous MRI screening round were correlated with lower re-attendance rates. Finally, a plurality of women mentioned an examination-related inconvenience as a reason to discontinue screening (39.1% and 34.8% in the second and third screening round, respectively).

**Conclusions:**

The willingness of women with dense breasts to re-attend an ongoing MRI screening study is high. However, emphasis should be placed on improving the MRI experience to increase the re-attendance rate if widespread supplemental MRI screening is implemented.

**Clinical relevance statement:**

For many women, MRI is an acceptable screening method, as re-attendance rates were high — even for screening in a clinical trial setting. To further enhance the (re-)attendance rate, one possible approach could be improving the overall MRI experience.

**Key Points:**

• *The willingness to re-attend in an ongoing MRI screening study is high.*

• *Pain, discomfort, and anxiety in the previous MRI screening round were related to lower re-attendance rates.*

• *Emphasis should be placed on improving MRI experience to increase the re-attendance rate in supplemental MRI screening.*

**Supplementary Information:**

The online version contains supplementary material available at 10.1007/s00330-024-10685-9.

## Introduction

Women with dense breasts have an increased risk of breast cancer compared to women who have more fatty breasts [1]. Moreover, the sensitivity of mammography is lower among women with dense breasts due to the masking effect of the dense breast tissue [1–3]. As a result, more tumours are missed in women with dense breasts at mammographic screening, resulting in an increased interval cancer rate; interval cancers are those detected in between screening rounds. Interval cancers are generally more aggressive as they are typically larger, grow faster, and spread more quickly than cancers detected at screening, and they are often found at a later or symptomatic stage [4, 5]. Therefore, the Dense Tissue and Early Breast Neoplasm Screening (DENSE) trial investigated the effectiveness of supplemental magnetic resonance imaging (MRI) on reducing interval cancer rates in women with dense breasts (ClinicalTrials.gov number: NCT01315015) [6]. The results of the first screening round of the DENSE trial showed that adding MRI screening to biennial mammography resulted in significantly fewer interval cancers than if mammography was used alone [7].

In a previous study, we investigated the attendance rate in the first MRI round and the reasons for non-participation [8]. Fifty-nine percent of the women invited for supplemental MRI screening participated in the first round. Most mentioned reasons for non-participation were MRI-related inconveniences, such as claustrophobia, and/or self-reported contraindications, personal reasons, or anxiety regarding the result of supplemental screening. For a breast cancer screening program to be effective, it is important that women attend on a regular basis [9]. To inform the discussion about implementing MRI screening for women with extremely dense breasts, it is important to know whether they re-attend after one or more MRI screening rounds, and if not, why. Here, we present the re-attendance rates in subsequent screening rounds of the DENSE trial and reasons given by participants to discontinue screening during subsequent screening rounds. Knowledge of these MRI re-attendance rates and reasons for discontinuation in subsequent screening rounds could facilitate efforts to improve MRI screening uptake and experience.

## Materials and methods

### Study design and participants

The Dutch Minister of Health, Welfare and Sport, who was advised by the Health Council of the Netherlands (2011/2019 WBO, The Hague, The Netherlands), approved the DENSE trial on November 11, 2011. All participants provided written informed consent.

The DENSE trial is embedded within the Dutch population-based digital mammography screening program (age 50–75) and consists of three biennial screening rounds. The study design and outcomes of the first and second rounds have been described previously [6, 7, 10]. Women were eligible for the DENSE trial if they had extremely dense breasts (grade 4 or d) as measured with Volpara version 1.5 (Volpara Health Technologies) and if they had a negative mammography result (Breast Imaging Reporting and Data System [BI-RADS] category 1 or 2).

All MRI examinations were performed on 3.0-T MRI systems with the macrocyclic gadolinium-based contrast agent gadobutrol (0.1 mmol/kg body weight) (Gadovist; Bayer AG). Details on the full screening MRI protocol have been described previously [6].

### Workflow screening rounds DENSE trial

Between December 2011 and January 2016, 4783 women were randomised to the intervention arm and participated in the first screening round of the DENSE trial. Women with a breast cancer diagnosis, an age outside the age range of the screening program (> 75 years) or women who passed away or moved abroad during or after the previous screening round, were not invited for subsequent screening rounds. All other women were invited again for mammographic screening 2 years after the previous (first or second) MRI round; women who participated in this mammographic screening and again had a negative (‘normal’) mammography result were invited for the next MRI round. Women who were referred for diagnostic work-up as a result of the mammographic screening were excluded from the corresponding MRI round (not invited).

### Calculation of the re-attendance rate

We assessed the re-attendance rate as follows: the numerator consisted of all women who participated in the second (or third) MRI round of the DENSE trial. The denominator consisted of all women who were invited for the second (or third) MRI round.

As a sensitivity analysis, we used a different denominator consisting of all women who were invited for the second (or third) MRI round but also the women who had actively unsubscribed for further participation in the DENSE trial before they were invited, and women who had declined their invitation for mammography. We performed this sensitivity analyses because a woman’s decision to decline the mammography invitation could have been influenced by their previous MRI experience. To further elaborate this hypothesis, we analysed the previous MRI experiences in attendance subgroups (participants, non-participants of MRI, non-participants of mammography and MRI). Information on reasons for declining mammography screening invitations was not available.

### Participant characteristics related to re-attendance

We described participant characteristics of women who re-attended and those who did not.

Participants completed a self-report questionnaire about demographic, reproductive and lifestyle factors, and their (family) medical history. We collected information about postal codes from the data available from the Dutch mammography program to classify socioeconomic status (SES).

### Factors related to MRI screening experience

MRI-related (serious) adverse events ((S)AEs) were reported directly after the MRI and were self-reported by women within 30 days after the MRI examination when applicable. An MRI screen-specific items questionnaire was administered 2 days after each MRI examination to assess pain, discomfort, and anxiety experienced during the MRI examination [11]. A false-positive finding was defined as a positive MRI result (BI-RADS 3, 4, or 5) without a diagnosis of breast cancer.

### Collection of self-reported reasons for discontinuation

Women were able to discontinue participation in the DENSE trial at any time. In this case, they were asked to provide one or more reasons for discontinuation. Subsequently, all reasons were registered, and we classified them into the following categories: MRI-related inconveniences and/or self-reported contraindications, anxiety regarding the outcome, personal reasons, practical reasons, burden too high, or reasons related to surveillance (e.g. already under active surveillance) [8]. We classified concerns around gadolinium retention in the brain as a reason for discontinuation under MRI-related inconveniences and/or self-reported contraindications [12, 13]. From March 2020 onwards, women were also able to provide ‘not wanting to go to the hospital due to the COVID-19 pandemic’ as a reason, which we categorised as a practical reason. When women declined the invitation due to a later acquired contraindication, we classified this as an MRI-related inconvenience and/or self-reported contraindication. Reasons were classified by two authors, and in case of disagreement, consensus was reached upon assessment of a third author.

### Data analyses

The outcome for the analyses was screening re-attendance as defined previously. We reported characteristics and previous MRI experience as proportions (percentage) of women in the category that re-attended. We reported the means with standard deviation for normally distributed continuous variables.

We examined differences between participants and non-participants using Pearson’s chi-squared tests for categorical variables and *t*-tests for normally distributed continuous variables. We calculated *p*-values for trend. Additionally, we determined whether factors were associated with re-attendance, by fitting logistic regression models adjusted for age to calculate odds ratios (ORs) with corresponding 95% confidence intervals (CIs). An OR above 1 indicates that women are more likely to drop-out, thus less likely to re-attend, in the next MRI screening round.

We summarised reasons to discontinue screening using descriptive statistics for all women who actively unsubscribed for further participation in the trial after the first or second round or who declined the invitation for the subsequent MRI screening round.

Calculations were based on the data collected until 2020–10-06.

We performed all analyses using RStudio software (RStudio, version 1.3.1093).

## Results

### Re-attendance rates

**Fig. 1:**
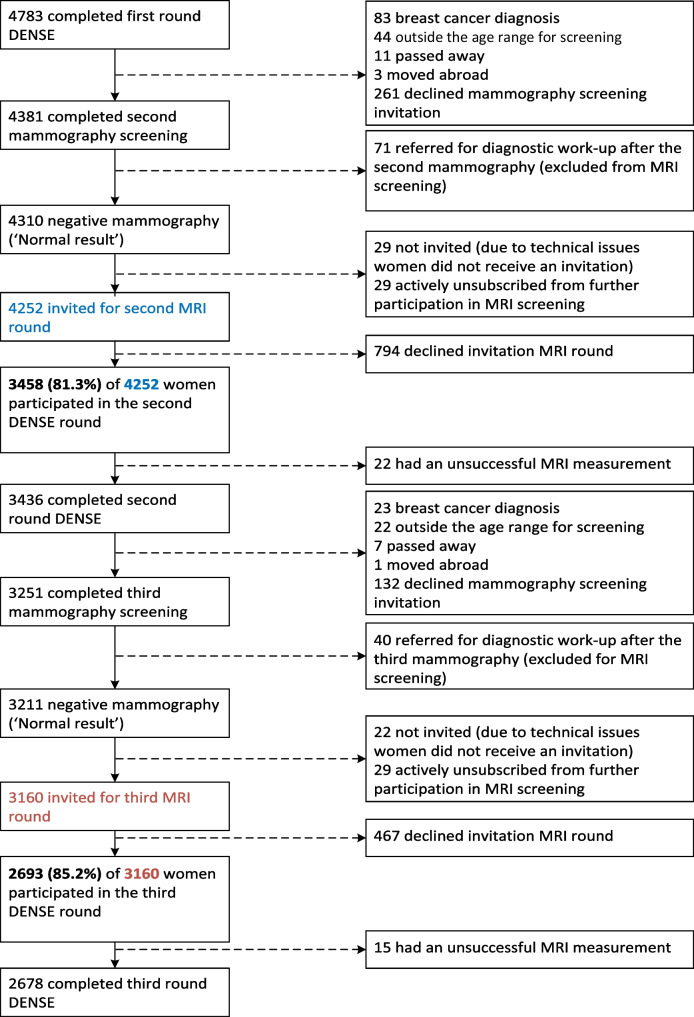
Flowchart of re-attendance in the DENSE trial. Second round: 4252 (100%) women were invited for the second MRI screening. Of these, 3458 (81.3%) women participated. Third round: 3160 (100%) women were invited for the third MRI screening. Of these, 2693 (85.2%) women participated

Figure 1 shows the flowchart of participation in the first, second, and third rounds of the DENSE trial. In the first MRI round, 4783 women participated. Between the first round and before the invitations for the second MRI round, women were excluded for various reasons (e.g. moved abroad, passed away, outside age range). A total of 3458 women participated in the second MRI screening round. The denominator of the re-attendance rate was 4252, which was the number of women who were invited for the second MRI screening round. This resulted in a re-attendance rate in the second DENSE MRI round of 81.3% (3458/4252).

Between the second and third MRI screening rounds, women were excluded for various reasons (e.g. moved abroad, outside age range). A total of 2693 women participated in the third MRI screening round. The denominator of the re-attendance rate was 3160, which was the number of women who were invited for the third MRI screening round. This resulted in a re-attendance rate in the third MRI round of 85.2% (2693/3160).

As a sensitivity analysis, we calculated the re-attendance rate with the same numerator but a different denominator (including the women who declined the mammography screening invitation and the women who actively unsubscribed for further participation in MRI screening). This resulted in a re-attendance rate in the second MRI round of 76.1% (3458 / (4252 + 261 + 29 = 4542)) and a re-attendance rate in the third MRI round of 81.1% (2693 / (3160 + 132 + 29 = 3321)). Related to this analysis, we created subgroups to check the hypothesis that a previous MRI experience could influence the decision to decline the next mammography invitation (see Supplemental Tables 1 and 2). Of the women who experienced very much anxiety during the first MRI round, 29% (5/17) declined the next mammography invitation, compared to 26% (153/586) of the women who experienced no anxiety during the previous MRI round (*p* = 0.04). Of the women who had a false-positive result in the first MRI round, 43% (46/108) declined the next mammography invitation, compared to 22% (215/976) of the women who experienced no anxiety during the previous MRI round (*p* < 0.01). These women, who did not attend mammographic screening, were not invited for MRI screening. Similar results were observed for the third round, although less profound (Supplemental Table 2).

### Study population characteristics

After adjusting for age, nine characteristics were statistically significantly associated with re-attendance in the second screening round (Table 1). Older women (70–74 years) were more likely to drop-out than younger women (50–54 years) (OR, 2.09; 95% CI, 1.28–3.31). Women with a BMI between 18.5–24.9 and 25–30 (kg/m^3^) were less likely to drop-out than women with a BMI below 18.5 (OR, 0.57, 95% CI, 0.41–0.80; OR, 0.50, 95% CI, 0.33–0.76, respectively.). Women who had a higher education were less likely to drop-out than women who had a lower education (OR, 0.52; 95% CI, 0.30–0.94). Women who were currently employed were less likely to drop-out than women who were not employed (OR, 0.49; 95% CI, 0.37–0.67). Women who currently smoked were more likely to drop-out than women who never smoked (OR, 1.59; 95% CI, 1.26–1.99). Women with a moderate alcohol consumption were less likely to drop-out than women with no alcohol consumption (OR, 0.59; 95% CI, 0.47–0.75). Women were more likely to drop-out with increasing pain during the previous MRI round (OR^little^, 1.95; OR^moderate^, 2.64; OR^very much^, 8.04). Women were more likely to drop-out with increasing discomfort during the previous MRI round (OR^little^, 1.49; OR^moderate^, 2.63; OR^very much^, 4.73). Women were more likely to drop-out with increasing anxiety during the previous MRI round (OR^little^, 1.89; OR^moderate^, 4.72; OR^very much^, 2.97). The results of the third screening round were comparable to that of the second screening round; however, the associations between age or education and re-attendance were less profound.

**Table 1:** Participant characteristics of women who were invited and re-attended versus women who were invited and discontinued in the second and third MRI screening round of the DENSE trial

	2nd screening round					3rd screening round				
	Total *N* = 4252^a^ (100%)	Yes *N* = 3458 (81.3%)	*p*-value^b^	*p*-trend	Age-adjusted OR (95% CI)	Total *N* = 3160^a^ (100%)	Yes *N* = 2693 (85.2%)	*p*-value^b^	*p*-trend	Age-adjusted OR (95% CI)
Age in years			< 0.01	< 0.01				0.20	0.09	
50–54	2194	1835/2194 (84%)			1.00^c^	1683	1450/1683 (86%)			1.00^c^
55–59	860	719/860 (84%)			0.98 (0.78–1.22)	671	587/671 (87%)			0.89 (0.68–1.16)
60–64	534	437/534 (82%)			1.13 (0.87–1.45)	405	339/405 (84%)			1.21 (0.89–1.62)
65–69	339	250/339 (74%)			1.79 (1.35–2.35)**	227	190/227 (84%)			1.21 (0.82–1.75)
70–74	94	67/94 (71%)			2.09 (1.28–3.31)**	41	32/41 (78%)			1.75 (0.78–3.57)
*Missing*	*231*	*150*				*133*	*95*			
Body mass index (BMI) (kg/m^3^)			< 0.01	< 0.01				< 0.01	0.21	
< 18.5	191	138/191 (72%)			1.00	123	91/123 (74%)			1.00
18.5–24.9	3310	2738/3310 (83%)			0.57 (0.41–0.80)**	2516	2176/2516 (86%)			0.45 (0.30–0.70)**
25–30	425	360/425 (85%)			0.50 (0.33–0.76)**	326	277/326 (85%)			0.52 (0.31–0.86)*
> 30	44	37/44 (84%)			0.63 (0.26–1.38)	29	24/29 (83%)			0.62 (0.19–1.64)
*Missing*	*281*	*185*				*166*	*125*			
Medical centre^d^			0.10					0.06		
UMCU	1408	1179/1408 (84%)			1.00	1078	908/1078 (84%)			1.00
Radboud UMC	377	309/377 (82%)			1.03 (0.75–1.41)	283	233/283 (82%)			1.06 (0.73–1.51)
AvL	214	175/214 (82%)			1.10 (0.73–1.62)	156	136/156 (87%)			0.79 (0.46–1.29)
ASZ	456	356/456 (78%)			1.30 (0.98–1.71)	317	270/317 (85%)			0.89 (0.61–1.26)
MUMC	283	225/283 (80%)			1.20 (0.85–1.68)	206	167/206 (81%)			1.12 (0.74–1.66)
JBZ	494	403/494 (82%)			1.12 (0.84–1.47)	369	329/369 (89%)			0.61 (0.41–0.89)*
VUmc	429	337/429 (79%)			1.33 (1.00–1.77)	312	263/312 (84%)			0.96 (0.66–1.36)
ZGT	591	474/591 (80%)			1.21 (0.93–1.57)	439	387/439 (88%)			0.62 (0.43-0.88)**
Travel distance to MRI centre in kilometres, mean (SD)^e^			0.78^f^	NA				0.30^f^	NA	
	24.5 (12.7)	24.4 (12.7)			1.00 (0.99–1.01)	24.4 (12.7)	24.5 (12.7)			1.00 (1.00–1.00)
Marital status			0.30	0.70				0.12	0.69	
Married	2817	2334/2817 (83%)			1.00	2140	1842/2140 (86%)			1.00
Unmarried but living with partner	334	273/334 (82%)			1.22 (0.80–1.82)	247	209/247 (85%)			1.12 (0.77–1.60)
Divorced	264	210/264 (80%)			1.26 (0.91–1.71)	192	158/192 (82%)			1.33 (0.89–1.95)
Unmarried, single	442	368/442 (83%)			1.09 (0.81–1.46)	337	299/337 (89%)			0.80 (0.55–1.13)
Widowed	144	111/144 (77%)			1.03 (0.78–1.35)	101 (100%)	81/101 (80%)			1.47 (0.86–2.39)
*Missing*	*251*	*162*				*153*	*104*			
Highest education			< 0.01	< 0.01				0.18	0.02	
Primary school	63	45/63 (71%)			1.00	41	33/41 (80%)			1.00
Lower vocational or lower secondary general education	1069	845/1069 (79%)			0.72 (0.41–1.30)	769	642/769 (83%)			0.83 (0.39–1.97)
Intermediate vocational or higher secondary education	1226	1014/1226 (83%)			0.62 (0.36–1.13)	933	806/933 (82%)			0.68 (0.32–1.62)
Higher vocational education or university education	1646	1396/1646 (85%)			0.52 (0.30–0.94)*	1278	1113/1278 (86%)			0.63 (0.30–1.50)
*Missing*	*248*	*158*				*139*	*99*			
Socio-economic status^g^			0.20	0.05				> 0.9	0.53	
Q1 (lowest SES)	624	491/624 (79%)			1.00	442	375/442 (85%)			1.00
Q2	973	785/973 (81%)			0.84 (0.65–1.09)	726	613/726 (84%)			1.05 (0.75–1.50)
Q3	1030	844/1030 (82%)			0.81 (0.62–1.05)	768	656/768 (85%)			1.01 (0.71–1.43)
Q4 (highest SES)	1616	1330/1616 (82%)			0.80 (0.63–1.02)	1216	1041/1216 (86%)			1.02 (0.75–1.42)
*Missing*	*9*	*8*				*8*	*8*			
Employment status			< 0.01	< 0.01				< 0.01	< 0.01	
No, never	239	171/239 (72%)			1.00	158	127/158 (80%)			1.00
No, but I used to	883	697/883 (79%)			0.61 (0.44–0.85)**	622	514/622 (83%)			0.85 (0.55–0.85)
Yes, I do	2884	2431/2884 (84%)			0.49 (0.37–0.67)**	2239	1950/2239 (87%)			0.61 (0.41–0.94)*
*Missing*	*246*	*159*				*141*	*102*			
Number of working hours/week, mean (SD)			< 0.01^e^	NA				< 0.01^e^	NA	
	18.3 (15.3)	18.9 (15.3)			0.99 (0.98–1.00)**	19.0 (14.2)	19.4 (15.1)			0.99 (0.98–1.00)**
*Missing*	*282*	*189*				*182*	*122*			
Smoking status			< 0.01	< 0.01				0.01	< 0.01	
Never	1673	1407/1673 (84%)			1.00	1289	1123/1289 (87%)			1.00
Former	1710	1414/1710 (83%)			1.05 (0.88–1.26)	1296	1114/1296 (86%)			1.08 (0.86–1.36)
Current	627	480/627 (77%)			1.59 (1.26–1.99)**	436	355/436 (81%)			1.53 (1.14–2.04)**
*Missing*	*242*	*157*				*139*	*101*			
Alcohol consumption^h^			< 0.01	< 0.01				< 0.01	< 0.01	
Never	450	339/450 (75%)			1.00	311	247/311 (79%)			1.00
Former	68	56/68 (82%)			0.64 (0.32–1.21)	52	44/52 (85%)			0.68 (0.28–1.45)
Moderate (< 14 glasses/week)	3081	2583/3081 (84%)			0.59 (0.47–0.75)**	2360	2030/2360 (86%)			0.62 (0.47–0.85)**
Excessive (> 14 glasses/week)	398	313/398 (79%)			0.76 (0.55–1.06)	288	263/288 (91%)			0.35 (0.21–0.57)**
*Missing*	*255*	*167*				*149*	*109*			
Exercise (Cambridge Physical Activity Index)^i^			0.14	0.03				0.30	0.97	
Inactive	168	133/168 (79%)			1.00	115	94/115 (82%)			1.00
Moderately inactive	700	560/700 (80%)			0.98 (0.65–1.50)	507	437/507 (86%)			0.72 (0.43–1.26)
Moderately active	1193	991/1193 (83%)			0.81 (0.55–1.23)	906	793/906 (88%)			0.65 (0.39–1.10)
Active	1821	1518/1821 (83%)			0.79 (0.54–1.19)	1404	1198/1404 (85%)			0.78 (0.48–1.31)
*Missing*	*370*	*256*				*228*	*171*			
Menarche age			0.30	0.44				0.40	0.93	
< 12 years old	314	255/314 (81%)			1.00	235	196/235 (83%)			1.00
12–13 years old	1764	1472/1764 (83%)			0.86 (0.63–1.18)	1355	1174/1355 (87%)			0.77 (0.54–1.14)
≥ 14 years old	1864	1520/1864 (82%)			0.97 (0.72–1.33)	1381	1181/1381 (86%)			0.85 (0.59–1.25)
*Missing*	*310*	*211*				*189*	*142*			
Menopausal status^j^			0.01	< 0.01				0.40	0.47	
Postmenopausal	2472	1975/2472 (80%)			1.00	1802	1532/1802 (85%)			1.00
Perimenopausal	1200	1017/1200 (85%)			0.90 (0.72–1.13)	944	820/944 (87%)			0.97 (0.74–1.28)
Premenopausal	407	350/407 (86%)			0.84 (0.60–1.16)	311	265/311 (85%)			1.13 (0.77–1.65)
*Missing*	*173*	*116*				*103*	*76*			
Parity			0.70	0.69				0.10	0.12	
Nulliparous	930	772/930 (83%)			1.00	698	615/698 (88%)			1.00
1 birth	467	380/467 (81%)			1.12 (0.84–1.50)	345	289/345 (84%)			1.44 (0.99–2.07)
2 or more births	2616	2152/2616 (82%)			1.06 (0.87–1.30)	1980	1690/1980 (85%)			1.28 (0.99–1.67)
*Missing*	*239*	*154*				*137*	*99*			
Breastfeeding history			0.20	> 0.9				0.60	0.68	
Ever, < 6 months	788	639/788 (81%)			1.00	577	488/577 (85%)			1.00
Ever, > 6 months	1576	1317/1576 (84%)			0.88 (0.71–1.11)	1221	1056/1221 (86%)			0.87 (0.66–1.15)
Never	1649	1348/1649 (82%)			0.96 (0.77–1.20)	1225	1050/1225 (86%)			0.91 (0.69–1.21)
*Missing*	*239*	*154*				*137*	*99*			
Breast biopsy history			0.54^ k^	0.45				0.03^ k^	0.02	
Never	3361	2777/3361 (83%)			1.00	2557	2212/2557 (87%)			1.00
Ever, but results unknown	625	505/625 (81%)			1.08 (0.86–1.34)	446	366/446 (82%)			1.39 (1.06–1.80)*
Atypical hyperplasia	19	16/19 (84%)			0.77 (0.18–2.35)	15	12/15 (80%)			1.54 (0.35–4.89)
*Missing*	*247*	*160*				*142*	*103*			
Breast cancer history 1st-degree relatives			0.04	0.04				0.07	0.07	
Yes	652	549/652 (84%)			1.00	501	440/501 (88%)			1.00
No/unknown	3600	2909/3600 (81%)			1.20 (0.96–1.52)	2659	2253/2659 (85%)			1.24 (0.93–1.66)
Breast cancer history 2nd-degree relatives			0.02	0.02				0.01	0.01	
Yes	1054	883/1054 (84%)			1.00	816	717/816 (88%)			1.00
No/unknown	3198	2575/3198 (81%)			1.11 (0.92–1.35)	2344	1976/2344 (84%)			1.26 (0.99–1.60)
History other cancers 1st-degree relatives (prostate-, ovarian-, colon cancer, and melanoma)			0.70	0.75				0.04	0.37	
Yes	1343	1096/1343 (82%)			1.00	1004	875/1004 (87%)			1.00
No/unknown	2909	2362/2909 (81%)			0.95 (0.81–1.13)	2156	1818/2156 (84%)			1.19 (0.96–1.49)
MRI experience during the previous round										
Reported perceived pain during previous MRI round			< 0.01^ k^	< 0.01				< 0.01^ k^	< 0.01	
Not	3342	2824/3342 (85%)			1.00	2461	2142/2461 (87%)			1.00
A little	313	231/313 (74%)			1.95 (1.48–2.55)**	200	159/200 (80%)			1.69 (1.15–2.44)**
Moderate	37	25/37 (68%)			2.64 (1.27–5.21)**	22	15/22 (68%)			3.00 (1.06–7.56)*
Very much	7	3/7 (43%)			8.04 (1.76–41.13)**	2	0/2 (0%)			NA^l^
*Missing*	*553*	*375*				*475*	*377*			
Reported perceived discomfort during previous MRI round			< 0.01	< 0.01				< 0.01	< 0.01	
Not	1324	1161/1324 (88%)			1.00	1069	944/1069 (88%)			1.00
A little	1834	1533/1834 (84%)			1.49 (1.21–1.85)**	1338	1162/1338 (87%)			1.20 (0.93–1.54)
Moderate	444	331/444 (75%)			2.63 (2.00–3.46)**	247	194/247 (79%)			2.09 (1.44–2.99)**
Very much	104	66/104 (63%)			4.73 (3.05–7.26)**	40	25/40 (63%)			4.19 (1.97–8.50)**
*Missing*	*594*	*367*				*466*	*368*			
Reported perceived anxiety during previous MRI round			< 0.01	< 0.01				< 0.01^ k^	< 0.01	
Not	2904	2493/2904 (86%)			1.00	2298	2013/2298 (88%)			1.00
A little	645	496/645 (77%)			1.89 (1.52–2.34)**	338	271/338 (80%)			1.75 (1.29–2.33)**
Moderate	112	66/112 (59%)			4.72 (3.15–7.02)**	38	25/38 (66%)			3.67 (1.80–7.14)**
Very much	40	28/40 (70%)			2.97 (1.44–5.79)**	10	6/10 (60%)			4.71 (1.20–16.58)*
*Missing*	*551*	*375*				*476*	*378*			
False-positive results at the previous MRI round			> 0.9	0.81				0.80	0.84	
No (true negatives)	3947	3210/3947 (81%)			1.00	3083	2628/3083 (85%)			1.00
Yes (false positives)	305	248/305 (81%)			1.08 (0.78–1.47)	77	65/77 (84%)			1.08 (0.53–1.98)

*OR*, odds ratio; *95% CI*, 95% confidence interval; * < 0.05, ** < 0.01

Interpretation OR: OR below 1 indicates that women were less likely to drop-out, thus more likely to re-attend, in the next MRI screening round

Characteristics are measured at baseline unless specified otherwise

^a^This total number of women represents the women that were invited for that particular screening round

^b^*p*-values are calculated using the chi-squared test

^c^Crude odds ratios were calculated for age

^d^Abbreviations: *UMCU*, University Medical Center Utrecht; Radboud UMC, Radboud University Medical Center; AvL, Antoni van Leeuwenhoek; ASZ, Albert Schweizer Hospital; MUMC, Maastricht University Medical Center; JBZ, Jeroen Bosch Ziekenhuis; VUmc, Amsterdam University Medical Center, location VUmc; ZGT, Ziekenhuisgroep Twente (Hospital group Twente)

^e^Calculated with postal codes

^f^*p*-value calculated with independent sample *T*-test

^g^Calculated with postal codes and the Neighbourhood Social Status Score of 2014 provided by The Netherlands Institute for Social Research

^h^According to the thresholds of the Dutch National Institute for Public Health and the Environment [23]

^i^Defined by the Cambridge Physical Activity Index (combination of self-reported weekly hours of cycling and sports with occupational physical activity). Women who were not (or no longer) employed were assigned to a level of occupational physical activity of 1 (being ‘inactive’) [24]

^j^Postmenopausal: if women were above or equal to the age of 60, reported a history of hysterectomy or bilateral oophorectomy, or reported zero periods within the last 12 months without the use of hormonal contraceptives. Premenopausal: if women reported regular periods (12–18 times in the last 12 months) without the use of hormonal contraceptives. Perimenopausal: all other women

^k^Fisher-exact test

^l^Number too low to calculate the odds ratio

### Reasons for discontinuation

Table 2 summarises the reasons for discontinuation for the women who either actively unsubscribed for further participation or who declined the invitation (*n* = 595 in the second round and *n* = 496 in the third round). In the second round, women who discontinued provided a total of 952 reasons.

**Table 2:** Reasons of discontinuation in the second and third screening round of the DENSE trial provided by women who actively dropped out or declined the invitation

Reason	Second round		Third round	
	Frequency (%)		Frequency (%)	
Number of women declining/opting out of MRI screening	**595**		**496**	
Total reasons given	**952**	**(100%)**	**566**	**(100%)**
MRI-related inconveniences and/or self-reported contraindications	**372**	**(39.1)**	**197**	**(34.8)**
*MRI specific*	**242**	**(25.4)**	**81**	**(14.3)**
Claustrophobia	71	(7.5)	32	(5.7)
Refusing MRI	6	(0.6)	3	(0.5)
Physical inability to adopt to/tolerate the right positioning for MRI	8	(0.8)	7	(1.2)
Contraindication for MRI; (self-reported); such as intracorporal metal	11	(1.2)	5	(0.9)
Unpleasant MRI experience	27	(2.8)	7	(1.2)
Painful MRI experience	53	(5.6)	8	(1.4)
Too much noise at MRI examination	66	(6.9)	19	(3.4)
*Contrast agent related*	**130**	**(13.7)**	**116**	**(20.5)**
Refusing/fear for needles	8	(0.8)	2	(0.4)
Refusing gadolinium or (self-reported) contraindication for gadolinium	54	(5.7)	61	(10.8)
Due to extra information letter on gadolinium health risks	62	(6.5)	47	(8.3)
Allergic reaction during MRI	6	(0.6)	3	(0.5)
GFR too low/creatinine too high	0	(0.0)	3	(0.5)
Anxiety caused by MRI examination	**79**	**(8.3)**	**33**	**(6.7)**
Emotional burden too high	66	(6.9)	19	(3.4)
Concerns about false positives or over-diagnostics	6	(0.6)	4	(0.7)
Aversion to hospitals or refusing any medical procedures	7	(0.7)	10	(1.8)
Practical reasons	**220**	**(23.1)**	**138**	**(24.4)**
Time constraints	116	(12.2)	45	(8.0)
The questionnaires	47	(4.9)	33	(5.8)
Travel related inconveniences	10	(1.1)	7	(1.2)
Other priorities	37	(3.9)	49	(8.7)
Financial concerns (costs possible additional interventions/diagnostics)	6	(0.6)	0	(0.0)
Impossible to schedule MRI appointment/no show	4	(0.4)	2	(0.4)
Not willing to come to the hospital due to the corona pandemic (March 2020) onwards)	0	(0.0)	2	(0.4)
Personal reasons	**158**	**(16.6)**	**107**	**(18.9)**
Other disease/health concerns	85	(8.9)	59	(10.4)
Low estimate of own risk (‘The regular screening program is sufficient for me’/ ‘I feel safe after one negative MRI’)	37	(3.9)	24	(4.2)
Inability to oversee consequences/aims of the study	0	(0.0)	0	(0.0)
Personal reasons without further explanation	17	(1.8)	15	(2.7)
Age	1	(0.1)	0	(0.0)
Not satisfied with the way being treated	18	(1.9)	9	(1.6)
Burden too high (without explanation)	**20**	**(2.1)**	**10**	**(1.8)**
Surveillance related	**8**	**(0.8)**	**4**	**(0.7)**
Already under active surveillance/recently referred before MRI	3	(0.3)	2	(0.4)
Made an appointment with my GP	0	(0.0)	0	(0.0)
Participation was discouraged by others/my GP	2	(0.2)	2	(0.4)
Aversion to extra prevention	2	(0.2)	0	(0.0)
Don’t want to participate in an RCT/research	1	(0.1)	0	(0.0)
Reason could not be classified	**4**	**(0.4)**	**5**	**(0.9)**
Reason not given or specified	**91**	**(9.6)**	**72**	**(12.7)**
No interest; not specified (‘I’m not interested anymore’)	75	(7.9)	60	(10.6)
No reason given	14	(1.5)	10	(1.8)
Died	2	(0.2)	2	(0.4)

*GP*, general practitioner; *GFR*, glomerular filtration rate; *RCT*, randomised controlled trial

Reasons for discontinuation reported by women actively dropping out of the DENSE trial or declining invitation for the DENSE trial in the second or third round. Women could give several reasons. The number of reasons in each category is presented and the percentages are calculated by dividing the number by the total number of reasons given in that round

Approximately 39.1% (372/952) of all provided reasons were MRI-related inconveniences and/or self-reported contraindications. MRI-related inconveniences can be subdivided into MRI-specific reasons (25.4%), such as claustrophobia and too much noise at the MRI examination, and contrast agent–related reasons (13.7%), such as refusing gadolinium. Moreover, 8.3% of all provided reasons to discontinue were related to anxiety caused by the MRI examination, 23.1% to practical reasons, and 16.6% to personal reasons. In the third screening round, women who discontinued provided a total of 566 reasons. Approximately 34.8% of all provided reasons were MRI-related inconveniences and/or self-reported contraindications. MRI-related inconveniences can be subdivided into MRI-specific reasons (14.3%), such as claustrophobia and too much noise at the MRI examination, and contrast agent–related reasons (20.5%), such as refusing gadolinium. Moreover, 6.7% of all provided reasons to discontinue were related to anxiety caused by the MRI examination, 24.4% to practical reasons, and 18.9% to personal reasons. The reasons to discontinue in the second or third rounds were similar.

## Discussion

In this study, we investigated the re-attendance in subsequent screening rounds of the DENSE trial. Among those invited, the re-attendance rates were high: 81.3% (3458/4252) in the second and 85.2% (2693/3160) in the third round of the DENSE trial. Women who did not re-participate, in both screening rounds: more often had a very low BMI (< 18.5); were less often employed; were more often current smokers; were less often moderate alcohol consumers; and more often perceived pain, discomfort, or anxiety during the previous MRI round. MRI-related inconveniences, specifically reasons such as claustrophobia and too much noise, were mentioned most frequently in both screening rounds as a reason not to continue screening.

We do not have much literature to compare our study with, as there are limited screening MRI studies with multiple screening rounds. Multiple studies address adherence in mammographic screening programs. Our results are in line with the results of a meta-analysis conducted by Damiani et al, who concluded that women with a higher educational status were more likely to adhere to mammographic breast cancer screening than women with a lower educational status [14].

Furthermore, we studied the reasons for discontinuing screening; MRI-specific inconveniences such as claustrophobia and too much noise at the MRI examination were most frequently mentioned reasons to discontinue in our study. This indicates that a prior unfavourable experience with breast MRI could have a negative impact on women’s willingness to re-attend in another screening round. In a previous paper on initial reasons for non-participation in the DENSE trial, MRI-specific inconveniences, with claustrophobia being the most frequently cited, were also most often given as reason to not participate [8]. A recent study conducted by Berg et al investigated patient preferences for contrast-enhanced mammography (CEM) versus MRI as supplemental screening options [15]. One frequently mentioned reason for preferring CEM over MRI was also claustrophobia. However, it should be noted that the effectiveness of CEM in a population-based screening setting merits further validation. A potential approach to reduce the concern about claustrophobia would be to offer an abbreviated form of MRI, in which women spend less time inside the MRI machine. Additionally, it is important to give the opportunity to women to communicate any concerns or potential discomforts with the medical staff before the MRI examination. They can provide guidance on how to manage or alleviate some of these discomforts. Finally, among the MRI-related inconveniences, contrast agent–related reasons were given, which likely would affect not only MRI, but also other contrast-enhanced techniques, including CEM. An alternative study that does not require contrast is ultrasound. However, due to limited incremental cancer detection yield of ultrasound, the European Society of Breast Screening (EUSOBI) recommends this technique only in situations where MRI screening is unavailable [16].

We found no difference in re-attendance in both MRI screening rounds between women who had a false-positive result in the previous MRI screening round and women who had not. However, in the sensitivity analyses, we found that women who had a false-positive result in the previous MRI screening round less often participated in the subsequent mammographic screening round. Women who did not attend the mammographic screening did not receive an invitation for the next MRI screening round. This finding is in line with most previous studies that investigated the effect of a false-positive result on re-attendance in mammographic screening; women with a false-positive mammogram were less likely to re-attend and were more likely to delay their subsequent screening [17, 18]. However, some studies have found the opposite effect of a false-positive result [19]. In a previous paper, we showed that the false-positive rate decreased from 79.8/1000 screenings in the first MRI round to 26.3/1000 screenings in the second MRI round [10]. It is expected that the false-positive rate will decrease further during subsequent screening rounds. Thus, any adverse effects on attendance due to false positives are expected to decrease with incidence screening.

A major strength of this study is the large sample size. Moreover, the sample population is a good representation of the domain under study due to the multicentre design of the trial and its embedment in the national breast cancer screening program. A limitation, however, is that we do not have any direct information about ethnicity, since it is illegal to register ethnicity in The Netherlands. This makes it more difficult to extrapolate these results to other populations. Another limitation of the study is that we had no information on reasons for drop-out for women who did not return to the mammographic screening program. An unfavourable MRI screening experience in the preceding screening round might be a reason not to return to the mammographic breast cancer screening program.

Recently, the EUSOBI recommended MRI as a supplemental screening technique for women with extremely dense breasts, but the debate on its implementation in breast screening programs is ongoing [19]. From our study, we conclude that for many women MRI is an acceptable screening method, as re-attendance rates were high — even for screening in a clinical trial setting. To further increase (re-)attendance, one option could be to improve MRI experience. This could be accomplished by implementing the use of wide-bore MRI scanners, which might decrease feelings of claustrophobia [20, 21], or implementing abbreviated forms of MRI which reduce acquisition time and noise levels. Furthermore, the occurrence of false-positive MRI results could potentially be reduced in the future, by using prediction rules for false-positive outcomes based on patient and imaging characteristics and/or introducing machine learning methods that could better distinguish malignant from benign breast cancer lesions on an MRI scan [22]. Finally, awareness and better education about extremely dense breasts and supplemental screening might increase (re-)attendance.

## Supplementary Information

Below is the link to the electronic supplementary material.

Supplementary file1 (PDF 227 KB)

## Abbreviations

CEM: Contrast-enhanced mammography
CI: Confidence interval
DENSE trial: Dense Tissue and Early Breast Neoplasm Screening
EUSOBI: European Society of Breast Imaging
MRI: Magnetic resonance imaging
OR: Odds ratio
SAE: Serious adverse events
SES: Socioeconomic status

## Declarations

### Guarantor

The scientific guarantor of this publication is Carla van Gils.

### Statistics and biometry

No complex statistical methods were necessary for this paper.

### Study subjects or cohorts overlap

The trial participants and other trial outcomes have been described earlier:

1. Bakker MF, de Lange SV, Pijnappel RM et al (2019) Supplemental MRI screening for women with extremely dense breast tissue. *New England Journal of Medicine* *381*.22:2091–2102

2. De Lange SV, Bakker MF, Monninkhof EM et al (2018) Reasons for (non) participation in supplemental population-based MRI breast screening for women with extremely dense breasts. *Clinical Radiology* *73*.8:759-e1.

3. Veenhuizen SG, de Lange SV, Bakker MF et al (2021) Supplemental breast MRI for women with extremely dense breasts: results of the second screening round of the DENSE trial. *Radiology* *299*.2:278–286.

The outcomes related to participation in the second and third screening round of DENSE have not been published before.

### Methodology

• prospective

• randomised controlled trial

• multicentre study
