# Supplementary material for: Re-attendance in supplemental breast MRI screening rounds of the DENSE trial for women with extremely dense breasts
Source: Eur Radiol. 2024 Apr 19;34(10):6334–47. doi: 10.1007/s00330-024-10685-9 (PMC11399182; doi:10.1007/s00330-024-10685-9)
Supplement: Supplementary file 1 — Supplementary file1 (PDF 227 KB) [file 330_2024_10685_MOESM1_ESM.pdf]

Supplemental Table 1. MRI-experience during the first screening round per attendance subgroup

	Total (n=1084)	Participants second mammography & non-participants second MRI (n=823)	Non-participants second mammography & non-participants second MRI (n=261)	*p*-value[a]
**Reported perceived pain during previous MRI round**				0.39[b]
Not	713 (100%)	539 (76%)	174 (24%)	
A little	109 (100%)	86 (79%)	23 (21%)	
Moderate	14 (100%)	13 (93%)	1 (7%)	
Very much	6 (100%)	4 (67%)	2 (33%)	
Missing	*242*	*181*	*61*	
**Reported perceived discomfort during previous MRI round**				0.33
Not	233 (100%)	170 (73%)	63 (27%)	
A little	413 (100%)	315 (76%)	98 (24%)	
Moderate	149 (100%)	118 (79%)	31 (21%)	
Very much	48 (100%)	40 (83%)	8 (17%)	
Missing	*241*	*180*	*61*	
**Reported perceived anxiety during previous MRI round**				0.04[b]
Not	586 (100%)	433 (74%)	153 (26%)	
A little	185 (100%)	153 (83%)	32 (17%)	
Moderate	55 (100%)	49 (84%)	9 (16%)	
Very much	17 (100%)	12 (71%)	5 (29%)	
Missing	*241*	*179*	*62*	
**False positive results at the previous MRI round**				<0.01
No (true negatives)	976 (100%)	761 (78%)	215 (22%)	
Yes (false positives)	108 (100%)	62 (57%)	46 (43%)	

[a] *p*-values are calculated using the Chi-square test.
[b] *p*-value is calculated using the Fisher exact test due to low numbers.

Supplemental Table 2. MRI-experience during the second screening round per attendance subgroup				
	**Total (n=650)**	**Participants third mammography & non-participants third MRI (n=518)**	**Non-participants third mammography & non-participants third MRI (n=132)**	***p*-value[a]**
**Reported perceived pain during previous MRI round**				0.16[b]
Not	441 (100%)	357 (81%)	84 (19%)	
A little	49 (100%)	45 (92%)	4 (8%)	
Moderate	7 (100%)	7 (100%)	0 (0%)	
Very much	2 (100%)	2 (100%)	0 (0%)	
Missing	*151*	*107*	*44*	
**Reported perceived discomfort during previous MRI round**				0.16[b]
Not	171 (100%)	138 (81%)	33 (19%)	
A little	251 (100%)	202 (80%)	49 (20%)	
Moderate	61 (100%)	55 (90%)	6 (10%)	
Very much	16 (100%)	16 (100%)	0 (0%)	
Missing	*151*	*107*	*44*	
**Reported perceived anxiety during previous MRI round**				0.06[b]
Not	394 (100%)	318 (81%)	76 (19%)	
A little	86 (100%)	74 (86%)	12 (14%)	
Moderate	14 (100%)	14 (100%)	0 (0%)	
Very much	5 (100%)	5 (100%)	0 (0%)	
Missing	*151 (100%)*	*107*	*44*	
**False positive results at the previous MRI round**				0.29[b]
No (true negatives)	627 (100%)	502 (80%)	125 (20%)	
Yes (false positives)	23 (100%)	16 (70%)	7 (30%)	

[a] *p*-values are calculated using the Chi-square test.
[b] *p*-value is calculated using the Fisher exact test due to low numbers.
